# Combined Central Retinal Vein and Branch Retinal Artery Occlusion Post Intense Physical Activity

**DOI:** 10.7759/cureus.1600

**Published:** 2017-08-23

**Authors:** Mircea Coca, Nahom Tecle, Wendewessen Amde, Ankur Mehta

**Affiliations:** 1 Chicagoland Retinal Consultants; 2 Rush University Medical Center; 3 Vitreoretinal Specialists

**Keywords:** vein occlusion, artery occlusion, physical activity, dehydration, marathon, scotoma

## Abstract

We report a case of combined central retinal vein occlusion and branch retinal artery occlusion. A previously healthy 47-year-old male presented with decreased vision in the right eye after completing a half marathon. A fundus exam and retinal imaging revealed a combined central retinal vein and branch retinal artery occlusion.

In the present report, we review the literature and discuss the possible mechanisms behind combined retinal vessel occlusions.

To our knowledge, this is the first reported case of combined central retinal vein occlusion and branch retinal artery occlusion following intense exercise.

## Introduction

Combined central retinal vein occlusion (CRVO) and branch retinal artery occlusion (BRAO) rarely occur together and predominantly arise in patients over 60 years of age [1]. Fortunately, CRVOs and BRAOs are rare in young patients. These two types of ocular vascular obstructions are typically associated with similar systemic conditions, such as cardiovascular atherosclerotic disease, arterial hypertension, and diabetes mellitus. In addition, certain uveitic and vasculitic conditions, lymphoproliferative disorders, medications, trauma, as well as hypercoagulable syndromes, such as antiphospholipid syndrome and homocysteinemia, can lead to these occlusive events [2]. There have been few case reports in the literature of vascular occlusions in young patients following intense dehydration due to either fasting or intense exercise [2-4].

## Case presentation

**Figure 1:**
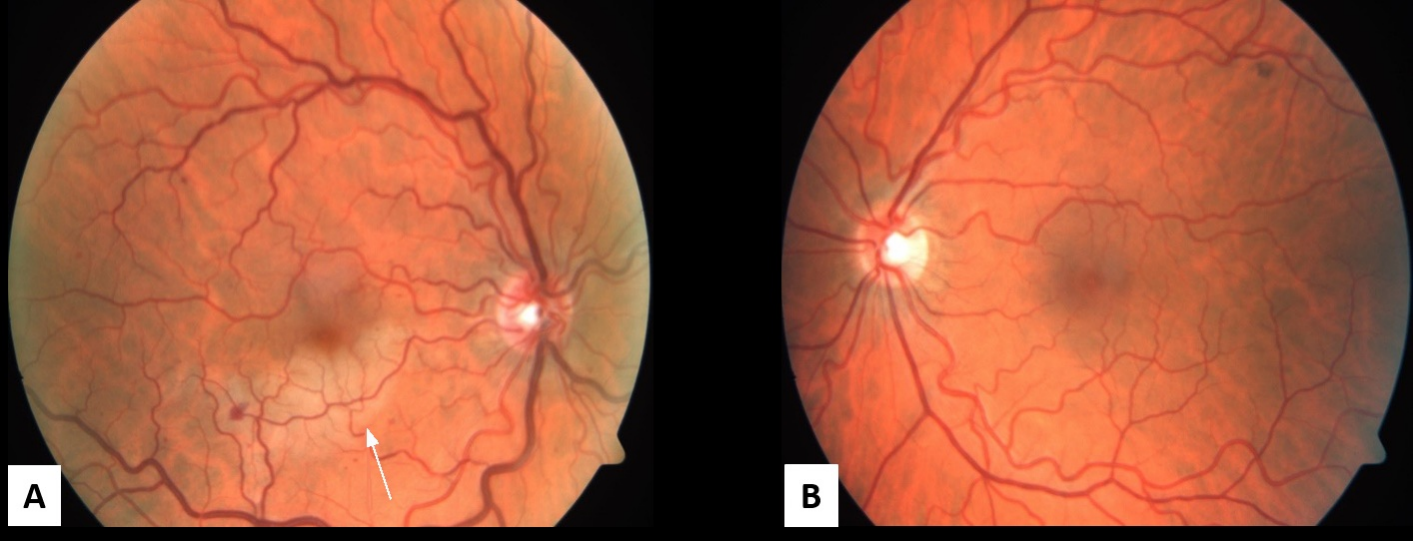
Fundus Photos A. The right eye fundus photo shows retinal whitening along the inferior arcade with scattered dot-blot hemorrhages (white arrow), dilated and tortuous vessels. B. The left eye fundus photo shows mild tortuous vessels with the rest of exam normal.

**Figure 2:**
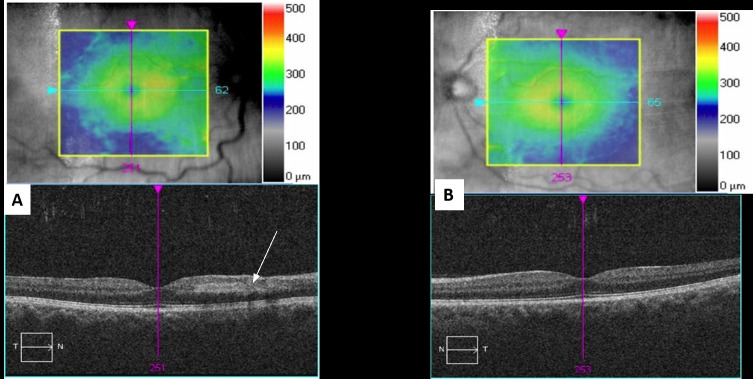
Optical Coherence Tomography Macula A. The right eye spectral-domain optical coherence tomography shows inner retinal hyper-reflectivity (white arrow) due to axoplasmic flow stasis along inferior arcade (seen near the optic nerve in this cut). B. The left eye spectral-domain optical coherence tomography shows a normal exam.

**Figure 3:**
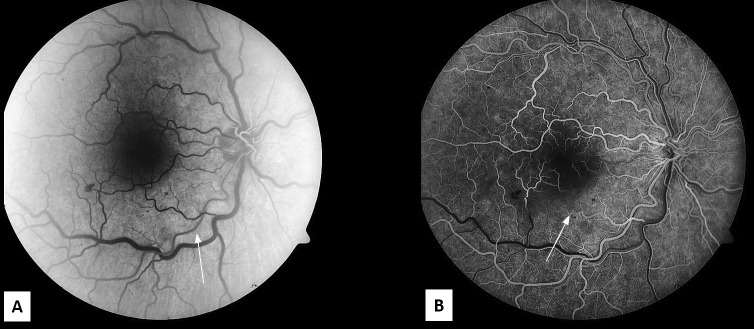
Fluorescein Angiogram A. The right eye fluorescein angiography in arterial phase at 26.7 s showing delayed transit time in the inferior arcade artery (white arrow). B. The early laminar flow venous phase at 39.8 s shows reduced perfusion in the inferior macula (white arrow).

A 47-year-old, previously healthy, male presented with a central scotoma in his right eye after running a half marathon. He denied any associated symptoms, including pain, photopsias, or floaters. A review of the systems was negative except for a family history of glaucoma (grandmother) and cataracts (grandmother). His best corrected visual acuity (BCVA) was 20/20 in both eyes and intraocular pressure was 24 mmHg in the right eye and 20 mmHg in the left eye. A fundus examination of the right eye revealed retinal whitening along the inferior arcade with scattered dot-blot hemorrhages near the macula and mid-periphery and dilated and tortuous veins (Figure 1A). The fundus exam of the left eye was normal (Figure 1B). The anterior segment slit lamp exam was normal in both eyes. The right eye spectral-domain optical coherence tomography (SD-OCT) was positive for inferior and nasal intraretinal fluid (IRF) with a foveal thickness of 298 microns (Figure 2A, 2B). Right eye fluorescein angiography (FA) was positive for a delayed arterial transit time in the inferior arcade artery consistent with branch retinal artery occlusion (Figure 3A, 3B). 

The dilated and tortuous veins, slightly delayed arteriovenous transit on fluorescein angiogram, and a few peripheral dot-blot hemorrhages of the right eye suggest that the patient had an incipient CRVO together with an inferior BRAO. The patient underwent a full hypercoagulable workup, which was negative. A carotid Doppler ultrasound was performed, and it did not show any significant narrowing or soft dislodging plaques. He was started on timolol bid in both eyes to decrease intraocular pressures.

At the one-month follow-up, his vision remained 20/20 bilaterally. The retina whitening and hemorrhages had resolved. He continued to note a scotoma in his right eye, which had improved since the initial visit. He was continued on timolol bid in both eyes and was advised not to perform endurance exercises.

## Discussion

Retinal vascular occlusions following intense exercise and dehydration are a rare occurrence. In the literature, there are few reports of isolated CRVO caused by intense exercise [2-3]. Our relatively young patient developed combined CRVO and BRAO soon after completing a half marathon. He had no previous medical or ocular history, and workup was negative for the known risk factors for vaso-occlusive diseases of the retina such as hypertension, diabetes, hyperlipidemia, hypercoagulable state, hyperviscosity state, atrial fibrillation, valve dysfunction, collagen vascular diseases, lymphoproliferative disorders, and hyperhomocysteinemia [1]. Hyperviscosity causes sluggish blood flow and a predisposition to coagulation in conditions including severe dehydration, hyperproteinemia, and dysproteinemia (e.g., macroglobulinemias, cold agglutinins), polycythemia, leukemia, and sickle cell disease [5]. The cause of the combined CRVO/BRAO in our patient is presumed to be due to a transient increase in plasma viscosity and hematocrit secondary to dehydration, leading to thrombotic events.

Different combinations of retinal artery and vein occlusion as a result of different etiologies have been reported in the literature [6]. These include combined CRVO/CRAO, CRVO/cilioretinal artery, CRVO/BRAO, and BRVO/BRAO. For combined CRVO/CRAO and CRVO/cilioretinal occlusion, where the site of the occlusion is within the optic nerve, the mechanism of the occlusion can be explained by the anatomical relationship between these vessels as they course through the optic nerve. The lamina cribrosa is a space within the optic nerve through which the retinal vein and artery pass to get to the intraocular space. The vascular congestion that follows the thrombotic or embolic occlusion of an artery or vein within the lamina cribrosa may result in a secondary occlusion of other vascular structures by direct compression. This is supported by D. Schmidt’s case series of 14 patients with acute unilateral visual loss due to combined retinal artery and venous occlusions. No emboli were found in any of these patients, while the cause of singular retinal artery occlusions in most patients is embolic [6].

However, the mechanism behind combined retinal vein and branch retinal artery occlusion is less intuitive. Two plausible mechanisms have been proposed to explain such combined occlusions. The first mechanism suggests the occluded vessel (vein or artery) compressing the unoccluded vessel at the site of an arteriovenous crossing. Retinal arteries and veins share a common adventitial sheath at crossing sites, and a distended vein or artery secondary to an occlusion may result in the compression of the normal vessel at these points. The second mechanism suggests that the occlusion initially takes place in the venous system [7]. Since the retinal circulation is a closed system, it is probable that a pressure increase and resistance to flow in the venous system could eventually backflow in the arterial circulation, decreasing the rate of arterial blood flow. Such stagnation of blood in the arterial system, especially in a person who is in a hypercoagulable state, may result in a secondarily retinal arterial occlusion. This mechanism could explain our patient’s findings, given his presumed hypercoagulable state from dehydration-induced hyperviscosity.

Red blood cells (RBCs) play a key role during exercise by transporting oxygen from the lungs to the tissues and delivering carbon dioxide to the lungs for expiration. Studies have shown that athletes tend to have a lower plasma viscosity and hematocrit (sports anemia) as well as less rigid RBCs as compared to normal individuals [8]. The lower baseline plasma viscosity may play a protective role and explain why exercise-induced retinal vascular occlusion is not a commonly occurring event in athletes who engage in vigorous physical activity on a regular basis. In addition to the lower baseline plasma viscosity, erythrocytes in athletes appear resistant to exercise-induced oxidative stress compared to erythrocytes in sedentary individuals [9]. Since erythrocyte oxidative stress has been shown to play a role in cell deformability in patients with retinal vein occlusion [10], having erythrocytes that are resistant to oxidative damage may play an additional protective role against exercise-induced retinal vascular occlusion in athletes.

Studies have shown hemorheological alterations in patients who develop exercise-induced central retinal vein thrombosis [11]. These patients could benefit from a standardized submaximal exercise-testing to look at the red cell aggregation and disaggregation threshold [11].

## Conclusions

We report the case of a young patient who developed combined CRVO and BRAO soon after completing a half marathon. We review the literature and discuss the possible mechanisms behind combined retinal vessel occlusions. To our knowledge, this is the first report of combined CRVO and BRAO following intense exercise.
